# Microbiological Evaluation of Upper Respiratory Tract Specimens in Patients with Plaque Psoriasis and Psoriatic Arthritis: A Retrospective Study

**DOI:** 10.3390/pathogens15090967

**Published:** 2026-09-11

**Authors:** Joanna Czerwińska, Ewelina Woźniak, Paulina Dobecka, Karolina Jamrozik, Dariusz Załuski, Agnieszka Owczarczyk-Saczonek

**Affiliations:** 1 Department of Dermatology, Sexually Transmitted Diseases and Clinical Immunology, School of Medicine, University of Warmia and Mazury in Olsztyn, 10-719 Olsztyn, Poland; baldygaewelina@gmail.com (E.W.); agnieszka.owczarczyk@uwm.edu.pl (A.O.-S.); 2 School of Medicine, University of Warmia and Mazury in Olsztyn, 10-719 Olsztyn, Poland; paulinadobecka@gmail.com (P.D.); jamrozik.a.karolina@gmail.com (K.J.); 3 Department of Plant Breeding and Bioresource Engineering, University of Warmia and Mazury in Olsztyn, 10-719 Olsztyn, Poland; dariusz.zaluski@uwm.edu.pl

**Keywords:** psoriasis, psoriatic arthritis, microbiological environment

## Abstract

Psoriasis is a chronic immune-mediated skin disease associated with systemic inflammation and multiple environmental triggers, including microbial colonization and infections. However, the relationship between upper respiratory tract microorganisms and the clinical characteristics of plaque psoriasis and psoriatic arthritis remains incompletely understood. This retrospective study analyzed 624 patients with plaque psoriasis, including mild and moderate-to-severe disease, and psoriatic arthritis who presented with upper respiratory tract symptoms and clinical signs suggestive of pharyngeal involvement. Upper respiratory tract swabs were evaluated using culture-based methods. Clinical and inflammatory parameters, including disease severity assessed using the Psoriasis Area and Severity Index (PASI), systemic inflammation assessed using C-reactive protein (CRP), body mass index (BMI), age, sex, and smoking status, were analyzed. All patients were evaluated prior to initiation of systemic antipsoriatic treatment. Overweight and obesity were prevalent and were associated with higher CRP levels across clinical groups. Culture-positive results were identified in 247 patients, yielding 275 isolates, while 377 patients had culture-negative results with physiological flora only. The distribution of the most frequently isolated microorganisms was broadly similar across clinical subgroups, with limited variation according to psoriasis severity or psoriatic arthritis status. Multivariable analysis identified CRP and PASI as the most consistent factors associated with culture positivity in selected subgroups, with evidence of an interaction between psoriasis severity and systemic inflammatory burden. No consistent associations were observed between specific microorganisms and clinical measures of disease severity. Overall, systemic inflammatory and metabolic parameters showed more consistent associations with culture-positive pharyngeal findings than individual culture-detectable microorganisms. Given the retrospective design, symptom-based inclusion, and absence of a contemporaneous control group, these findings should be interpreted as descriptive and associative rather than causal. Prospective controlled studies using standardized sampling and culture-independent methods are needed to clarify the clinical significance and temporal relationship between upper respiratory tract microbial findings and psoriasis activity.

## 1. Introduction

Psoriasis is a chronic immune-mediated inflammatory skin disease affecting approximately 2–3% of the global population. Its pathogenesis involves a complex interaction between genetic susceptibility and environmental triggers, including psychological stress, mechanical trauma, medications, alcohol consumption, tobacco use, and infections [1,2,3,4,5,6,7]. Among infectious triggers, streptococcal throat infections have been most consistently associated with the onset and exacerbation of psoriasis, particularly in genetically predisposed individuals such as carriers of the HLA-Cw6 allele [8]. Proposed mechanisms include immune cross-reactivity, T-cell activation, and amplification of Th17-mediated inflammatory pathways [9,10].

Psoriasis is increasingly recognized as a systemic inflammatory disease rather than a condition limited to the skin. Persistent activation of the IL-23/Th17 axis and other inflammatory pathways is associated with elevated circulating inflammatory mediators and may contribute to the development of metabolic and cardiovascular comorbidities. In particular, overweight and obesity are highly prevalent in patients with psoriasis and may further increase systemic inflammatory burden, as reflected by elevated C-reactive protein (CRP) levels. Thus, metabolic status, chronic inflammation, and disease severity may interact in shaping the clinical phenotype of psoriasis [6]. The relationship between psoriasis and susceptibility to infections is more complex. Patients with psoriasis may have altered susceptibility to infections as a result of disease-associated immune dysregulation, comorbidities, and, in some patients, immunomodulatory treatment. Biologic and other systemic therapies can further modify infection risk, making it difficult to distinguish the effects of psoriasis itself from treatment-related susceptibility. Importantly, the present study included patients evaluated before initiation of systemic antipsoriatic treatment, thereby reducing the potential confounding effect of systemic therapy on upper respiratory tract microbiological findings. Whether patients with plaque psoriasis have distinct patterns of upper respiratory tract colonization remains insufficiently defined. Conversely, persistent systemic inflammation and immune dysregulation in psoriasis may influence host susceptibility to microbial colonization, suggesting a potentially bidirectional relationship between inflammatory disease activity and microbial exposure [11,12]. Beyond streptococcal infection, other microorganisms, including Staphylococcus aureus and Candida albicans, have been reported in patients with psoriasis and may be relevant to local or systemic immune responses, although their clinical significance in chronic plaque psoriasis remains less well established [2,3,4,13,14,15,16].

While the association between streptococcal infection and guttate psoriasis is well recognized, the relationship between chronic plaque psoriasis, psoriatic arthritis, and upper respiratory tract microbial colonization remains unclear. Existing studies report inconsistent findings regarding the presence of specific microorganisms and their association with disease severity or systemic inflammation [17,18,19,20]. Furthermore, the influence of common metabolic and lifestyle factors such as obesity and smoking on this relationship is not fully understood.

Given these uncertainties, characterization of upper respiratory tract microbiological findings in patients with psoriasis may help determine whether culture-detectable microorganisms are associated with systemic inflammation, disease severity, and metabolic or lifestyle factors.

This study aimed to evaluate upper respiratory tract cultures in patients with chronic plaque psoriasis and psoriatic arthritis and to investigate their associations with clinical parameters, including disease severity (Psoriasis Area and Severity Index, PASI), systemic inflammation (C-reactive protein, CRP), body mass index (BMI), age, sex, and smoking status.

## 2. Materials and Methods

### 2.1. Study Design and Population

A retrospective analysis was conducted on the medical records of 624 patients diagnosed with psoriasis before initiation of systemic therapy (Table 1). The cohort included 250 females and 374 males treated between 2018 and 2024 at the Dermatology Outpatient Clinic and the Department of Dermatology, Sexually Transmitted Diseases, and Clinical Immunology in Olsztyn, Poland. The study protocol was approved by the Bioethical Committee of the University of Warmia and Mazury in Olsztyn (Declaration No. 5/2026, 23 April 2026). Informed consent was not required for this retrospective study.

### 2.2. Clinical Inclusion and Exclusion Criteria

**Figure 1:**
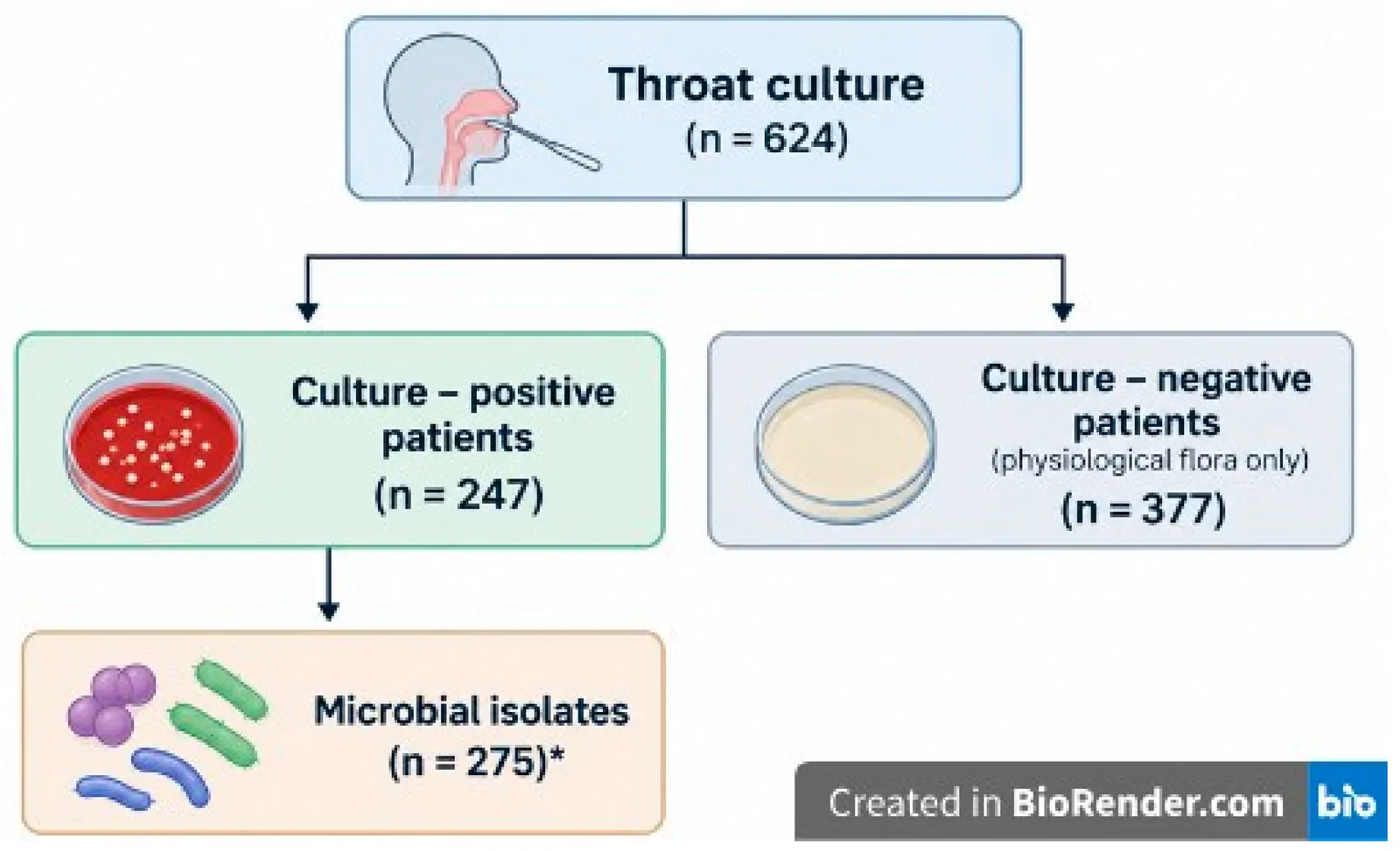
Flow diagram of patient selection and microbiological analysis. * Multiple microorganisms could be isolated from a single patient; therefore, the total number of isolates (*n* = 275) exceeds the number of patients with positive cultures (*n* = 247). Created in BioRender.com.

All included patients were referred for microbiological examination (Figure 1) because of suspected or reported symptoms suggestive of upper respiratory tract involvement, including sore throat, discomfort, scratchiness, and/or clinical signs such as pharyngeal erythema, exudates, tonsillar edema, or inflammatory changes of the posterior pharyngeal wall. Following clinical assessment, not all patients met the criteria for clinical pharyngitis. Thus, the study population comprised patients presenting with symptoms or clinical findings suggestive of upper respiratory tract involvement, whereas clinical pharyngitis was defined according to the criteria described below.

We excluded patients with clinical or laboratory signs of acute systemic infection at the time of medical evaluation to minimize confounding by acute infectious processes and focus on upper respiratory tract colonization or carrier states.

For the purposes of this study, acute systemic infection was operationally defined as at least one of the following criteria documented in the medical records: body temperature ≥ 38.0 °C; clinical symptoms of acute infection (e.g., chills, severe fatigue, or acute malaise); and/or markedly elevated inflammatory markers, defined as C-reactive protein (CRP) > 50 mg/L and/or leukocytosis > 15 × 10^9^/L.

Recent antibiotic or antifungal exposure outside the predefined exclusion criterion was not consistently documented and therefore could not be systematically accounted for. No exclusions were applied based on psoriasis severity, psoriatic arthritis status, smoking history, or body mass index.

### 2.3. Sample Collection and Microbiological Analysis

Microbiological swabs were collected from the posterior pharyngeal wall and tonsillar area using sterile techniques in fasting patients who had previously rinsed their oral cavity with sterile water. Sampling was performed with sterile swabs, with care taken to avoid contact with the tongue, uvula, or saliva contamination. In cases where visible tonsillar structures were present, material was obtained from the tonsillar surface; however, deep tonsillar crypt sampling was not systematically performed.

Specimens were transported within 2 h at room temperature to a certified external microbiology laboratory. Samples were cultured on 5% sheep blood agar (Columbia agar) and chocolate agar and incubated at 37 °C under aerobic and 5–10% CO_2_ conditions for 24–48 h. Fungal cultures were performed on chromogenic Candida agar with incubation up to 10 days.

Microorganisms were identified using routine microbiological procedures, with species identification and antimicrobial susceptibility testing performed using the VITEK 2 automated system (bioMérieux, Marcy-l’Étoile, France). No molecular diagnostic techniques (e.g., PCR) or serological assays (e.g., antistreptolysin O or antistreptococcal IgA/IgG) were performed. Viral pathogens were not systematically assessed; therefore, viral pharyngitis could not be excluded in patients with negative bacterial cultures.

The laboratory is a certified diagnostic facility operating under internal quality control standards. Antimicrobial susceptibility testing, where applicable, was performed according to standard clinical microbiology protocols.

Patients were stratified into three groups: mild psoriasis (mPsO)—BSA ≤ 10%, PASI ≤ 10, and DLQI ≤ 10; moderate-to-severe psoriasis (sPsO)—(BSA > 10% or PASI > 10) and DLQI > 10 [21]; and psoriatic arthritis (PsA)—diagnosed by a rheumatologist according to CASPAR criteria [22].

### 2.4. Additional Variables

Active smoking was defined as consumption of more than 5 cigarettes per day. This threshold was predefined to distinguish regular smoking from occasional or low-level tobacco exposure. Smoking status was recorded based on patient medical history.

We defined overweight and obesity according to World Health Organization (WHO) criteria as body mass index (BMI) ≥ 25 kg/m^2^ for overweight and ≥30 kg/m^2^ for obesity.

Clinical pharyngitis was defined as the presence of upper respiratory tract symptoms (e.g., sore throat, discomfort, or scratchiness) accompanied by objective clinical signs on examination (e.g., erythema, tonsillar exudates, or posterior pharyngeal wall inflammation). All patients meeting these clinical criteria underwent upper respiratory tract swab collection. Culture-positive results were defined as the isolation of at least one microorganism from the upper respiratory tract specimen, whereas culture-negative results indicated that only physiological flora was identified.

### 2.5. Post-Diagnostic Management

Following microbiological assessment, patients received treatment guided by antibiotic susceptibility testing (antibiogram) when clinically indicated. Data on selected potential confounding factors, including diabetes status, glycemic control (e.g., HbA1c), prior or current use of systemic or topical immunosuppressive or biologic therapies, recent antibiotic or antifungal treatment, corticosteroid use, and detailed oral or dental health status, were not consistently available in the retrospective medical records and therefore were not included in the analysis.

### 2.6. Statistical Analysis

All analyses were performed using STATISTICA 13.3 (TIBCO Software Inc., San Ramon, CA, USA, 2017; Statistica data analysis software system, version 13.). The distribution of continuous variables was assessed using the Shapiro–Wilk test. As most variables, including PASI, CRP, and BMI, were non-normally distributed, results are presented as medians with interquartile ranges (IQRs) or means with standard deviation (SD), as appropriate based on distribution characteristics.

For group comparisons, non-parametric tests were used, including the Mann–Whitney U test for two-group comparisons and the Kruskal–Wallis test for comparisons involving more than two groups. Associations between continuous variables (PASI, CRP, BMI, and age) were assessed using Spearman’s rank correlation coefficient.

To assess potential independent predictors of pharyngitis, multivariable logistic regression models were constructed, including PASI, CRP, BMI, and interaction terms where appropriate. Results are reported as odds ratios (ORs) with 95% confidence intervals (CIs). Additional covariates, including age, sex, and smoking status, were explored in sensitivity analyses where sample size allowed. Subgroup analyses were performed using stratified analyses.

Statistical significance was defined as *p* < 0.05. No formal correction for multiple comparisons was applied; therefore, subgroup analyses and their corresponding effect estimates should be interpreted cautiously as exploratory and hypothesis-generating rather than confirmatory. For each analysis, the number of available observations is reported, and missing observations were excluded from the respective analysis.

## 3. Results

### 3.1. Study Population Characteristics

A total of 624 patients were retrospectively analyzed, including patients with mild plaque psoriasis (mPsO; *n* = 233), moderate-to-severe plaque psoriasis (sPsO; *n* = 317), and psoriatic arthritis (PsA; *n* = 74). Overall, 68% (*n* = 424) of patients were classified as overweight or obese. No significant differences in age or sex distribution were observed among the three groups (*p* > 0.05).

Among men, CRP concentrations were highest in patients with PsA (12.46 ± 29.67 mg/L), compared with those with mPsO and sPsO (4.90 ± 9.62 and 6.66 ± 15.75 mg/L, respectively; *p* = 0.0347) (Table 2).

Overall, 68% (*n* = 424) of patients were classified as overweight or obese (OO; BMI ≥ 25 kg/m^2^), with a prevalence of 62% among women and 75% among men. Patients in the OO group were significantly older than those with BMI < 25 kg/m^2^ in both sexes (women: 54.29 ± 11.33 vs. 35.29 ± 16.62 years, *p* < 0.0001; men: 47.23 ± 13.93 vs. 39.46 ± 15.96 years; *p* < 0.0001). CRP levels were significantly higher in the OO group than in patients with BMI < 25 kg/m^2^ in both women and men (women: 6.04 ± 11.68 vs. 3.94 ± 10.51 mg/L, *p* < 0.0005; men: 6.77.27 ± 11.11 vs. 3.06 ± 3.42 mg/L, *p* < 0.0001). A weak but statistically significant positive correlation was observed between BMI and age (r = 0.246, *p* < 0.05).

### 3.2. Pharyngitis Prevalence and Subgroup Distribution

Among all patients, 249 (39.6%) were classified as having pharyngitis according to study criteria based on clinical symptoms and positive upper respiratory tract culture results. Patients were stratified according to microbiological culture results into culture-positive and culture-negative (physiological flora) groups for comparative analyses.

#### 3.2.1. Characteristics of the Mild Psoriasis (mPsO) Population

In the mPsO group, pharyngitis was identified in 38% of patients. A weak positive correlation was observed between BMI and age in this group (r = 0.2354, *p* < 0.05). In the group of patients with physiological flora, obese women were significantly older than normal-weight women (*p* = 0.002). Among men in the same group, CRP levels were significantly higher in obese compared with normal-weight participants (*p* = 0.042) (Table 3).

#### 3.2.2. Characteristics of the Moderate-to-Severe Psoriasis (sPsO) Population

In the sPsO group, pharyngitis was identified in 40% of patients. No significant association was observed between age and pharyngitis status. BMI showed positive correlations with age (r = 0.3249, *p* < 0.05), PASI (r = 0.1373), and CRP (r = 0.2311, *p* < 0.05). Obese women were significantly older than normal-weight women in both study groups (*p* = 0.019 and *p* = 0.0006). CRP levels were significantly higher in obese compared with normal-weight women in both groups (*p* = 0.023 and *p* = 0.012), as well as in obese compared with normal-weight men (*p* = 0.006 and *p* = 0.011) (Table 4).

#### 3.2.3. Characteristics of the Psoriatic Arthritis (PsA) Patient Population

In the PsA group, pharyngitis was identified in 43% of patients. Overweight was highly prevalent in this group (90% of women and 81% of men). No significant association was observed between overweight status and the frequency of pharyngitis (Table 5).

### 3.3. Multivariate Logistic Regression Analysis

Multivariate logistic regression identified a model that included PASI, CRP, BMI, and the interaction between psoriasis severity and systemic inflammation (PASI × CRP). In women with severe psoriasis, CRP was the only statistically significant predictor of pharyngitis (OR = 1.22, *p* < 0.05). PASI and BMI were not significant contributors. In men with severe psoriasis, both PASI (OR = 1.07 per unit increase, *p* < 0.05) and CRP (OR = 1.12 per unit increase, *p* < 0.05) were significant predictors of pharyngitis. The interaction between psoriasis severity and systemic inflammation was also significant, indicating modification of the PASI effect by CRP levels. BMI showed a positive trend that did not reach statistical significance (*p* = 0.0873).

In women with mild psoriasis, PASI (OR ≈ 1.58 per unit increase) and CRP were significant predictors of pharyngitis. BMI was inversely associated with pharyngitis. A significant interaction between psoriasis severity and systemic inflammation was observed. In men with mild psoriasis, the regression model did not identify significant predictors of pharyngitis (Table 6).

### 3.4. Characteristics of the Smoker Population

Among the study population, 204 patients were active smokers (>5 cigarettes/day), including 108 patients with positive upper respiratory tract cultures (29 women and 79 men). Smoking showed a weak negative correlation with BMI (r = –0.0985, *p* < 0.05) and a weak positive correlation with CRP (r = 0.1243, *p* < 0.05). No significant correlations were observed between smoking and PASI, disease stage, or age. In overweight patients with sPsO, a weak positive correlation between smoking and PASI was observed (r = 0.099, *p* < 0.05).

### 3.5. Logistic Regression Analysis

Multivariate logistic regression analysis identified a model including PASI, CRP, BMI, and the PASI × CRP interaction as the best-fitting model for the prediction of pharyngitis. In women with severe psoriasis, CRP was the only significant predictor of pharyngitis (OR = 1.22, *p* < 0.05), while PASI and BMI were not significant. In men with severe psoriasis, both PASI (OR = 1.07 per unit increase, *p* < 0.05) and CRP (OR = 1.12 per unit increase, *p* < 0.05) were significant predictors of pharyngitis. The PASI × CRP interaction was also significant. BMI showed a non-significant trend (*p* = 0.0873). In women with mild psoriasis, PASI and CRP were significant predictors of pharyngitis, while BMI showed an inverse association. In men with mild psoriasis, the regression model did not identify significant predictors of pharyngitis.

### 3.6. Qualitative Analysis of Upper Respiratory Tract Cultures

Qualitative analysis of positive upper respiratory tract cultures with species identification revealed similar distributions across psoriasis subgroups. In patients with PASI ≤ 10, the most frequently isolated organisms were *Streptococcus pyogenes* (41%) and *Streptococcus viridans* (21%). *Klebsiella pneumoniae* was detected in 3% of cases, while *Candida albicans* was identified in 10% of patients. In patients with PASI > 10, *Streptococcus viridans* (38%) and *Streptococcus pyogenes* (21%) predominated. Mixed cultures were observed in this group. In patients with psoriatic arthritis, the microbial profile included *Streptococcus pyogenes* (23%), *Streptococcus viridans* (15%), *Klebsiella pneumoniae* (15%), and *Candida albicans* (23%). No statistically significant associations were observed between isolated microorganism species and BMI, sex, or smoking status (Table 7).

## 4. Discussion

This retrospective study evaluated clinical, metabolic, and microbiological characteristics in 624 patients with plaque psoriasis and psoriatic arthritis, focusing on upper respiratory tract culture findings and their associations with systemic inflammatory and disease-related parameters. Given the exploratory nature of the analyses and the multiple subgroup comparisons, the findings should be interpreted cautiously as hypothesis-generating rather than confirmatory. Overall, metabolic status and systemic inflammation, reflected primarily by BMI and CRP, showed more consistent associations with clinical and inflammatory parameters than specific culture-based microbiological findings did. In contrast, no consistent associations were identified between individual culture-detectable microorganisms and psoriasis severity.

Overweight and obesity were highly prevalent in the study population and were associated with higher CRP levels, supporting a relationship between metabolic status and systemic inflammation in patients with psoriasis. However, the observed associations were generally weak, suggesting that metabolic status may represent a disease modifier rather than a direct determinant of psoriasis severity. Smoking showed only weak associations with BMI and CRP and was not consistently associated with disease severity, in line with previous reports describing complex and heterogeneous relationships between smoking, metabolic factors, and psoriasis activity.

Microbiological cultures of the upper respiratory tract demonstrated a predominance of Streptococcus spp. and Candida albicans across the clinical subgroups, with limited variation between mild psoriasis, moderate-to-severe psoriasis, and psoriatic arthritis. These findings are descriptive and reflect culture-based detection of colonizing or potentially pathogenic organisms within a symptomatic psoriasis cohort [23,24,25]. Importantly, no consistent associations were identified between specific microorganisms and disease severity, BMI, sex, or smoking status. The relatively similar culture profiles across clinical subgroups suggest that culture-detectable upper respiratory tract microorganisms were not consistently associated with clinical disease stratification in this cohort. However, these findings should not be interpreted as evidence that upper respiratory tract microorganisms are unrelated to psoriasis pathogenesis. Culture-based methods detect only a subset of microorganisms and do not characterize the broader microbial community, microbial abundance, or functional interactions [26,27]. Moreover, because all patients in the primary cohort had symptoms or clinical findings suggestive of upper respiratory tract involvement, the present study cannot determine whether the observed culture patterns differ from those in asymptomatic individuals or the general population. The association between upper respiratory tract findings and psoriasis is biologically plausible, particularly given the established relationship between streptococcal exposure and psoriasis exacerbation [1,5,6,7,8,9,11,12,13,14,15,16]. A prospective study of patients with chronic plaque psoriasis reported that sore throat and the isolation of β-haemolytic streptococci (Lancefield groups A, C, and G) were more frequent in patients with psoriasis than in controls, and psoriasis exacerbation was observed specifically among patients in whom streptococci were isolated following the onset of sore throat [24]. These findings support a temporal association between streptococcal throat infection and disease exacerbation. In contrast, our retrospective cross-sectional study assessed patients at a single clinical time point and was not designed to evaluate subsequent psoriasis exacerbations following pharyngeal infection. Accordingly, although Streptococcus spp. were frequently detected, we did not observe a consistent association between the presence of an individual culture-detectable microorganism and contemporaneous disease severity. The clinical significance of pharyngeal culture findings may also depend on the distinction between active infection and microbial carriage. In a recent multicenter study of pediatric patients with new-onset or worsening psoriasis, evaluation for pharyngeal and anogenital infections was recommended for consideration, particularly in patients presenting with disease onset or exacerbation [28]. Importantly, only 24% of patients with a positive pharyngeal culture were symptomatic, suggesting that some positive cultures may represent bacterial carriage rather than clinically apparent infection [28]. Although the pediatric population and clinical setting differed from those of our adult cohort, these observations are consistent with our finding that individual culture-detectable microorganisms were not consistently associated with psoriasis severity.

The present data likewise do not establish a causal relationship between specific microorganisms and psoriasis activity. Detection of a microorganism in a throat specimen cannot, by itself, distinguish active infection from colonization or carriage. This distinction is especially relevant for Streptococcus pyogenes and Candida spp., for which colonization may occur in the absence of clinically significant infection [29,30,31,32]. Furthermore, the clinical significance of streptococcal detection and treatment in psoriasis remains uncertain. A systematic review of antistreptococcal interventions in guttate and chronic plaque psoriasis identified only five randomized trials involving 162 participants, of whom only 23 had a streptococcus-positive throat culture [33]. The available evidence regarding systemic antibiotics and tonsillectomy was rated as very low quality, and the authors concluded that the efficacy and safety of antistreptococcal interventions remained uncertain [33]. Similarly, in a study of 115 patients with mild-to-moderate psoriasis, streptococcal throat infection was more frequent among patients with psoriasis than among healthy controls; however, adjunctive systemic antibiotic treatment in patients with active streptococcal infection was not associated with a greater reduction in PASI compared with psoriasis patients without evidence of active infection receiving standard topical treatment [34]. Together, these observations suggest that, although streptococcal pharyngeal infection may represent a clinically relevant trigger in selected patients, its detection and treatment do not necessarily translate into a predictable change in psoriasis severity.

Our study extends the existing literature by evaluating a large adult cohort with chronic plaque psoriasis and psoriatic arthritis and by examining pharyngeal culture findings in relation not only to disease severity but also to systemic inflammation, metabolic status, smoking, age, and sex. The broadly similar distribution of culture-detectable microorganisms across clinical subgroups and the absence of consistent associations between individual microorganisms and disease severity suggest that the relationship between pharyngeal microbial findings and chronic plaque psoriasis is more complex than the presence or absence of a particular organism. The multivariable analyses provide an additional perspective on the relationships between psoriasis severity, systemic inflammation, and culture positivity. In men with moderate-to-severe psoriasis, both PASI and CRP were significantly associated with culture positivity, and the significant association between PASI and culture positivity differed according to CRP levels suggests that the association between psoriasis severity and culture positivity may vary according to the level of systemic inflammatory activity. In women with moderate-to-severe psoriasis, CRP was the only factor significantly associated with culture positivity, suggesting that systemic inflammatory activity may be more closely related to culture positivity than psoriasis severity alone in this subgroup. A significant interaction between psoriasis severity and systemic inflammation was also observed in women with mild psoriasis, although the pattern of associations differed between strata. Collectively, these findings suggest potential heterogeneity in the relationships between psoriasis severity, systemic inflammation, and pharyngeal culture positivity according to sex and disease severity. However, the stratified analyses and multiple comparisons were exploratory, no formal adjustment for multiple testing was performed, and some subgroup sizes were limited. Therefore, these findings should be considered hypothesis-generating rather than confirmatory and require validation in larger prospective studies. The high prevalence of overweight and obesity in the present cohort is consistent with the well-established association between psoriasis, psoriatic arthritis, and metabolic comorbidities [35]. The positive association between BMI and CRP observed in our cohort further supports a relationship between excess body weight and systemic inflammatory burden. Nevertheless, the relatively weak correlation between BMI and age, together with the observational nature of the study, does not allow these relationships to be interpreted as causal. Rather, the findings support the concept that metabolic status may act as a modifier of the inflammatory milieu associated with psoriasis. Smoking showed weaker and less consistent associations with inflammatory and clinical parameters. Although smoking was associated with CRP and, in the overweight subgroup with moderate-to-severe psoriasis, showed a weak association with PASI, the low correlation coefficients and potential influence of unmeasured confounders limit the clinical interpretation of these findings [36,37,38]. These observations should therefore be regarded as supportive rather than definitive evidence of an association between smoking and inflammatory or disease-related parameters. An additional consideration is the possibility of a bidirectional relationship between upper respiratory tract microbial findings and psoriasis activity. Systemic inflammation associated with psoriasis may potentially influence host susceptibility to microbial colonization or infection, while microbial exposure or infection-related immune activation may potentially influence psoriasis activity. However, given the retrospective cross-sectional design, the absence of longitudinal follow-up, and the lack of a contemporaneous control group, our findings cannot establish the directionality of such relationships or determine whether the microorganisms identified contributed to psoriasis activity or primarily reflected colonization in an already inflamed host. Prospective longitudinal and controlled studies are therefore needed to clarify the temporal relationship between upper respiratory tract microbial findings and psoriasis exacerbations.

## 5. Limitations

Several limitations should be acknowledged. First, the retrospective design and symptom-based inclusion criteria may have introduced selection bias and preclude causal inference. The absence of a contemporaneous control group, including healthy individuals and patients with psoriasis without upper respiratory tract symptoms, limits the ability to determine whether the observed microbial profiles differ from those of the general population or are specific to psoriasis.

Second, the microbiological assessment was based exclusively on culture-dependent methods, which provide only a partial representation of the upper respiratory tract microbiota and do not allow the detection of viral pathogens. Therefore, viral pharyngitis could not be excluded, particularly in patients with negative bacterial cultures. The sampling strategy may also have affected the detection of *Streptococcus pyogenes*, as tonsillar crypts were not systematically sampled, and serological markers of previous streptococcal exposure were not available.

Third, some potentially relevant confounding factors, including diabetes and glycemic control, detailed treatment history, and previous antimicrobial exposure, were not consistently available in the retrospective records. This limited our ability to fully assess factors that may influence CRP levels and upper respiratory tract colonization, particularly by *Candida* spp. Residual confounding by other unmeasured clinical factors therefore cannot be excluded.

Finally, multiple subgroup comparisons and exploratory regression analyses were performed without formal correction for multiple testing, increasing the risk of type I error. Some subgroup analyses may also have been underpowered. Therefore, subgroup-specific associations should be considered hypothesis-generating and require confirmation in prospective studies with appropriate control groups and standardized clinical and microbiological assessments.

## 6. Conclusions

Overweight and obesity were highly prevalent among patients with psoriasis and psoriatic arthritis and were associated with higher CRP levels, supporting an association between excess body weight and systemic inflammatory burden. Smoking showed weaker and less consistent associations with inflammatory and clinical parameters. Culture-based analysis revealed broadly similar upper respiratory tract microbial profiles across psoriasis subtypes, with no consistent associations between specific microorganisms and psoriasis severity or psoriatic arthritis status. In contrast, systemic inflammatory parameters, particularly CRP, and disease severity assessed by PASI showed more consistent associations with culture-confirmed pharyngitis in selected subgroups, including an interaction between psoriasis severity and systemic inflammation. Overall, these findings suggest that host-related inflammatory and metabolic factors may be more relevant to pharyngeal findings than the presence of specific culture-detectable microorganisms in this symptomatic psoriasis cohort. Prospective controlled studies are needed to clarify the temporal and clinical significance of upper respiratory tract microbial findings in psoriasis.

## Figures and Tables

**Table 1:** Patient population’s characteristic (mean values ± SD; median); F—females; M—males; PASI—Psoriasis Area and Severity Index; BMI—body mass index; CRP—C-reactive protein.

		F (*n* = 250)	M (*n* = 374)
Age (years)	mean ± SD	46.9 ± 19.1	45.2 ± 10.8
	median	49	44
BMI	mean ± SD	27.9 ± 6.5	28.3 ± 13.3
	median	26.93	27.92
PASI	mean ± SD	12.4 ± 4.3	15.5 ± 4.4
	median	11.2	14.4
CRP (mg/L)	mean ± SD	5.3 ± 8.7	6.8 ± 12.9
	median	1.98	1.85

**Table 2:** Characteristics of the studied population by gender, age (years), body mass index—BMI (kg/m^2^), Psoriasis Area and Severity Index—PASI, and C-reactive protein—CRP (mg/L) (mean values ± SD; median and min/max). * indicates statistically significant differences of CRP between all study groups at *p* < 0.05; F—females, M—males; mPsO—mild plaque psoriasis, sPsO—moderate-to-severe plaque psoriasis, and PsA—psoriatic arthritis.

		Age (Years)	BMI	PASI	CRP (mg/L)
Mild psoriasis (mPsO)—PASI ≤ 10					
F (*n* = 108)	mean ± SD	48.52 ± 19.84	27.99 ± 8.20	5.96 ± 3.49	6.19 ± 15.79
	median (min/max)	51 (13/85)	28.28 (16.01/61)	6 (0.4–15)	1.5 (0.6/115.6)
M (*n* = 125)	mean ± SD	45.66 ± 19.69	27.27 ± 4.26	6.83 ± 4.85	4.90 ± 9.62
	median (min/max)	46.5 (18/92)	26.87 (19.3/39.8)	6.05 (1.2/12.6)	1.25 (0.6/48.4)
Moderate/severe psoriasis (sPsO)—PASI > 10					
F (*n* = 117)	mean ± SD	46.21 ± 19.24	28.86 ± 7.17	15.81 ± 6.01	4.76 ± 7.16
	median (min/max)	45 (18/83)	26.72 (18.6–50.5)	16.85 (16–36)	2.3 (0.6/45.2)
M (*n* = 200)	mean ± SD	44.77 ± 16.50	29.02 ± 5.02	17.79 ± 6.42	6.66 ± 15.75
	median (min/max)	43 (18/86)	28.39 (25.1/46.3)	17.3 (16.8/34.4)	2.5 (0.6/157)
Psoriatic arthritis (PsA)					
F (*n* = 25)	mean ± SD	52.40 ± 14.49	31.29 ± 9.30	14.33 ± 9.99	7.17 ± 12.73
	median (min/max)	53.5 (21/76)	28.46 (20.3/61)	14 (1.2/36)	2.3 (0.6/45.2)
M (*n* = 49)	mean ± SD	48.70 ± 12.54	28.95 ± 5.35	17.14 ± 7.29	12.46 ± 29.67 *
	median (min/max)	48 (22/70)	27.93 (18.5/41.8)	17.1 (4.4/34.2)	3.4 (0.6/157.2)

**Table 3:** Relationships between age (years), Psoriasis Area and Severity Index (PASI), C-reactive protein (CRP, mg/mL) (mean values ± SD; median and min/max) and body mass index (BMI): 18.5–24.9—normal weight (N); 25.0 and above—overweight/obesity (OO) and the occurrence of pharyngitis in the mild psoriasis (mPsO) patient group (*n* = 233). * indicates statistically significant differences in age between normal-weight and obese women in the non-pharyngitis group (*p* = 0.002). ** indicates a statistically significant difference in CRP levels between obese and normal-weight men in the culture-negative (physiological flora) group (*p* = 0.042); F—females, M—males.

			Age (Years)	PASI	CRP (mg/L)
	Culture-positive (*n* = 88)				
F (*n* = 45)	mean ± SD	OO 52% (*n* = 23)	56.33 ± 19.9	5.57 ± 2.95	5.95 ± 8.08
	median (min/max)		57 (18/76)	5.4 (0.2/15.8)	3 (0.4/8.9)
	mean ± SD	N 48% (*n* = 22)	30.31 ± 10.09	6.20 ± 2.52	3.66 ± 12.34
	median (min/max)		30 (15/65)	6.8 (4.3/9.8)	1.5 (0.2–78.4)
M (*n* = 43)	mean ± SD	OO 65% (*n* = 28)	47.34 ± 14.0	6.8 ± 1.70	9.9 ± 15.40
	median (min/max)		50 (7/75)	6.7 (4.6/9.6)	1.27 (0.3/48.4)
	mean ± SD	N 35% (*n* = 15)	34.57 ± 16.31	7.08 ± 2.10	1.4 ± 2.07
	median (min/max)		19 (15/65)	7 (4/8)	0.61 (0.5/1.26)
	Culture-negative—physiological flora (*n* = 145)				
F (*n* = 74)	mean ± SD	OO 64% (*n* = 47)	55.47 ± 19.12 *	5.81 ± 3.94	6.55 ± 17.91
	median (min/max)		60 (16/84)	5.9 (1.2/11)	2.35 (0.1/115.6)
	mean ± SD	N 36% (*n* = 27)	39.21 ± 12.24	5.1 ± 2.44	3.16 ± 4.69
	median (min/max)		30 (15/85)	6 (3.2/9.3)	0.7 (0.1/18.3)
M (*n* = 71)	mean ± SD	OO 65% (*n* = 46)	51.85 ± 14.18	6.3 ± 5.25	3.7 ± 5.46 **
	median (min/max)		55 (7/92)	5.65 (1.2/32.6)	3.05 (0.1/47.7)
	mean ± SD	N 35% (*n* = 25)	40.22 ± 16.96	7.03 ± 2.68	2.62 ± 4.68
	median (min/max)		34 (23/63)	7.6 (2.9/9.6)	0.5 (0.2/21.59)

**Table 4:** Relationships between age (years), Psoriasis Area and Severity Index (PASI), C-reactive protein (CRP, mg/mL) (mean values ± SD; median and min/max), body mass index (BMI): 18.5–24.9—normal weight (N); 25.0 and above—overweight/obesity (OO), and the occurrence of pharyngitis in the severe psoriasis (sPsO) patient group (*n* = 317). * indicates a statistically significant difference in age between obese and normal-weight women in both study groups (*p* =0.019 and *p* = 0.0006). ** indicates a statistically significant difference in CRP levels between obese and normal-weight women in both study groups (*p* = 0.023 and *p* = 0.012). *** indicates a statistically significant difference in CRP levels between obese and normal-weight men in both study groups (*p* = 0.006 and *p* = 0.011); F—females, M—males.

			Age (Years)	PASI	CRP (mg/L)
	Culture-positive (*n* = 127)				
F (*n* = 40)	mean ± SD	OO 51% (*n* = 21)	51.55 ± 13.82 *	17.86 ± 5.56	5.12 ± 6.04 **
	median (min/max)		54 (21/81)	14.8 (16.1/27.4)	4.16 (0.72/25.3)
	mean ± SD	N 49% (*n* = 19)	33.47 ± 11.28	15.72 ± 7.26	3.65 ± 3.87
	median (min/max)		32 (16/63)	14.9 (10/25)	0.7 (0.3/28.85)
M (*n* = 87)	mean ± SD	OO 78% (*n* = 68)	43.35 ± 10.84	19.74 ± 7.56	5.42 ± 6.10 ***
	median (min/max)		44.5 (7/84)	16.2 (14.6/36.4)	2.07 (0.3/157)
	mean ± SD	N 22% (*n* = 19)	41.22 ± 15.59	17.54 ± 5.83	3.81 ± 5.80
	median (min/max)		35 (15/73)	18.3 (14/33.7)	1.05 (0.3/61.59)
	Culture-negative—physiological flora (*n* = 190)				
F (*n* = 65)	mean ± SD	OO 71% (*n* = 46)	53.60 ± 19.35	16.54 ± 2.32	6.29 ± 1.03 **
	median (min/max)		54 (7/83)	15.7 (13.6/36)	2.8 (0.1/45.2)
	mean ± SD	N 29% (*n* = 19)	37.85 ± 13.08	15.99 ± 1.87	2.56 ± 0.76
	median (min/max)		30 (16/45)	18.2 (15.2/34.8)	0.7 (0.1/11.2)
M (*n* = 125)	mean ± SD	OO 80% (*n* = 100)	47.72 ± 15.39	17.8 ± 6.22	9.12 ± 19.03 ***
	median (min/max)		46 (4/92)	16.3 (11.2/34)	3 (0.1/47.7)
	mean ± SD	N 20% (*n* = 25)	40.41 ± 16.83	16.35 ± 6.22	7.76 ± 45.85
	median (min/max)		37 (18/72)	16.7 (16.9/27.4)	1.1 (0.2/29)

**Table 5:** Relationships between age (years), Psoriasis Area and Severity Index (PASI), C-reactive protein (CRP, mg/mL) (mean values ± SD; median and min/max), body mass index (BMI): 18.5–24.9—normal weight (N); 25.0 and above—overweight/obesity (OO), and the occurrence of pharyngitis in the psoriatic arthritis (PsA) patient group (*n* = 74); F—females, M—males.

			Age (Years)	PASI	CRP (mg/L)
	Culture-positive (*n* = 32)				
F (*n* = 11)	mean ± SD	OO 90% (*n* = 9)	52.36 ± 14.85	13.52 ± 10.77	8.91 ± 12.86
	median (min/max)		54 (19/53)	23.2 (8.4/19)	15.2 (3.1/38.38)
	mean ± SD	N 10% (*n* = 2)	44.98 ± 17.45	14.37 ± 2.28	5.43 ± 1.17
	median (min/max)		43 (37/63)	11.52 (10.25/14)	0.6 (0.6/0.6)
M (*n* = 21)	mean ± SD	OO 81% (*n* = 17)	58.19 ± 15.55	14.46 ± 8.38	17.14 ± 4.43
	median (min/max)		52 (26/70)	16.2 (10.8/34.2)	2 (0.4/157.8)
	mean ± SD	N 19% (*n* = 4)	59.54 ± 18.13	18.05 ± 6.16	3.10 ± 0.28
	median (min/max)		62.5 (56/69)	13 (8/18)	0.93 (0.6/1.26)
	Culture-negative—physiological flora (*n* = 42)				
F (*n* = 14)	mean ± SD	OO 86% (*n* = 12)	47.5 ± 13.87	14.38 ± 9.82	6.05 ± 12.47
	median (min/max)		53.5 (30/76)	11.15 (12/36)	1.2 (0.5/45.2)
	mean ± SD	N 14% (*n* = 2)	48.65 ± 16.45	11.21 ± 1.42	3.82 ± 0.65
	median (min/max)		36 (21/51)	15.45 (5.2/25.7)	2.35 (1/3.7)
M (*n* = 28)	mean ± SD	OO 92% (*n* = 25)	62.32 ± 17.34	17.66 ± 8.46	7.24 ± 33.38
	median (min/max)		47 (30/64)	17.6 (4.4/34)	4.35 (0.1/27.5)
	mean ± SD	N 8% (*n* = 3)	53.17 ± 18.43	14.80 ± 5.63	4.35 ± 1.03
	median (min/max)		33 (22/56)	22.4 (13.7/22.6)	2.9 (2.8/3.3)

**Table 6:** Multivariable logistic regression analysis of factors associated with pharyngitis stratified by sex and psoriasis severity. N—sample size; const—model intercept; estimate—effect estimate; OR—odds ratio; OR 95% CI, 95% confidence interval for OR; *p*-value, statistical significance. Statistically significant effects are indicated where *p* < 0.05.

Group	N	Parameter	Estimate	OR	OR 95% CI (Lower)	OR 95% CI (Upper)	*p*-Value
PS severe_females	98	const	−1.71	0.18	0.02	1.96	0.1594
	98	PASI	0.03	1.03	0.94	1.14	0.5179
	98	CRP	0.20	1.22	1.01	1.48	0.0378
	98	BMI	−0.01	0.99	0.92	1.06	0.8112
	98	PASI × CRP	−0.01	0.99	0.99	1.00	0.1041
PS severe_males	193	const	−3.31	0.04	0.01	0.25	0.0007
	193	PASI	0.07	1.07	1.00	1.14	0.0389
	193	CRP	0.11	1.12	1.01	1.24	0.0313
	193	BMI	0.05	1.05	0.99	1.11	0.0873
	193	PASI × CRP	−0.01	0.99	0.99	1.00	0.0396
PS mild_females	56	const	1.31	3.72	0.05	263	0.5450
	56	PASI	0.45	1.58	1.03	2.41	0.0366
	56	CRP	0.53	1.70	1.10	2.63	0.0162
	56	BMI	−0.23	0.79	0.65	0.97	0.0229
	56	PASI × CRP	−0.07	0.93	0.87	1.00	0.0494
PS mild_males	53	const	−3.95	0.02	0.00	1.76	0.0863
	53	PASI	−0.12	0.88	0.70	1.12	0.2978
	53	CRP	−0.15	0.86	0.62	1.19	0.3573
	53	BMI	0.13	1.14	0.97	1.34	0.1018
	53	PASI × CRP	0.03	1.03	0.97	1.10	0.3082

**Table 7:** Proportion of Gram-positive and Gram-negative bacterial species and yeast-like fungi isolated from upper respiratory tract swabs in patients with mild psoriasis (mPsO, *n* = 88), moderate/severe psoriasis (sPsO, *n* = 127), and psoriatic arthritis (PsA, *n* = 32). Multiple organisms may have been isolated from a single patient; therefore, total counts refer to isolates (*n* = 275) rather than individuals (*n* = 247).

Microorganism	mPsO, PASI < 10 (*n* = 88)	sPsO, PASI > 10 (*n* = 127)	PsA (*n* = 32)	All Cases	Smokers (*n* = 58)	Non smokers (*n* = 189)
Yeast-like fungi						
*Candida albicans*	13 (10.2)	15 (12.3)	6 (23)	34 (12.4)	12 (14.6)	22 (11.4)
*Candida parapsilosis*	1 (0.8)	1 (0.8)	0 (0)	2 (0.7)	0 (0)	2 (1.0)
*Candida tropicalis*	1 (0.8)	1 (0.8)	1 (4.3)	3 (1.1)	0 (0)	3 (1.6)
*Candida krusei*	1 (0.8)	1 (0.8)	0 (0)	2 (0.7)	0 (0)	2 (1.0)
*Candida glabrata*	1 (0.8)	0 (0)	0 (0)	1 (0.4)	0 (0)	1 (0.5)
Gram-positive bacteria						
*Streptococcus pyogenes*	29 (22.8)	25 (20.5)	6 (23.1)	60 (21.8)	17 (20.7)	43 (22.3)
*Streptococcus viridans*	52 (40.9)	46 (37.7)	4 (15.4)	102 (37.1)	33 (40.2)	69 (35.8)
*Streptococcus pneumoniae*	2 (1.6)	2 (1.6)	0 (0)	4 (1.5)	1 (1.2)	3 (1.6)
*Streptococcus agalactiae*	0 (0)	2 (1.6)	0 (0)	2 (0.7)	0 (0)	2 (1.0)
*Staphylococcus epidermidis*	0 (0)	1 (0.8)	0 (0)	1 (0.4)	0 (0)	1 (0.5)
*Staphylococcus aureus*	10 (7.9)	11 (9.0)	2 (7.7)	23 (8.4)	10 (12.2)	13 (6.7)
*Micrococcus* sp.	1 (0.8)	1 (0.8)	0 (0)	2 (0.7)	0 (0)	2 (1.0)
Gram-negative bacteria						
*Moraxella catarrhalis*	3 (2.4)	3 (2.5)	0 (0)	6 (2.2)	3 (3.7)	3 (1.6)
*Haemophilus influenzae*	3 (2.4)	2 (1.6)	0 (0)	5 (1.8)	3 (3.7)	2 (1.0)
*Klebsiella pneumoniae*	4 (3.1)	6 (4.9)	4 (15.4)	14 (5.1)	2 (2.4)	12 (6.2)
*Escherichia coli*	3 (2.4)	4 (3.3)	3 (11.5)	10 (3.6)	0 (0)	10 (5.2)
*Pseudomonas aeruginosa*	2 (1.6)	0 (0)	0 (0)	2 (0.7)	0 (0)	2 (1.0)
*Enterobacter cloacae*	1 (0.8)	1 (0.8)	0 (0)	2 (0.7)	1 (1.2)	1 (0.5)
Total (*n* cases):	127 (100)	122 (100)	26 (100)	275 (100)	82 (100)	193 (100)

## Data Availability

The raw data supporting the conclusions of this article will be made available by the authors on request.
